# Multimodal Neuromonitoring During Pediatric Cardiac Surgery

**DOI:** 10.21470/1678-9741-2020-0713

**Published:** 2022

**Authors:** Jyrson Guilherme Klamt, Waynice Neiva de Paula Garcia, Mariana de Carvalho, Luis Vicente Garcia, Antonio Carlos Menardi

**Affiliations:** 1 Department of Orthopedics and Anesthesiology, Faculdade de Medicina de Ribeirão Preto, Universidade de São Paulo, Ribeirão Preto, São Paulo, Brazil.; 2 Department of Surgery and Anatomy, Hospital das Clínicas da Faculdade de Medicina de Ribeirão Preto, Universidade de São Paulo, Ribeirão Preto, São Paulo, Brazil.

**Keywords:** Neuroprotection, Child, Cardiac Surgical Procedures, Anesthesia, Electroencephalography, Near-infrared Spectroscopy

## Abstract

**Introduction:**

Neuromonitoring (electroencephalogram [EEG] and cerebral oximetry) is essential for appropriate anesthesia and neuroprotection assessment during pediatric cardiac surgery.

**Methods:**

We describe the intraoperative pediatric multimodal and multiparametric neuromonitoring pattern of the software system Neuron-Spectrum (Kandel®) that consists of continuous electroencephalogram (cEEG), spectral analysis, amplitude-integrated electroencephalogram (aEEG), depth of anesthesia monitor (NINDEX), and regional cerebral and somatic oximetry (near-infrared spectroscopy-INVOS™). A physiological algorithm for management using neuromonitoring and physiological data is also described.

**Results:**

Visual data examples are presented for interpretation of the cerebral perfusion and oxygenation, neurophysiological state, anesthesia depth, possible neurologic predictions, and identification of cerebral drug effects (EEG signature). Conclusion: The neuromonitoring model can be an effective tool for anesthesia control and to provide adequate cerebral oxygenation during surgery.

**Table:** 

Abbreviations, acronyms & symbols			
aEEG	= Amplitude-integrated electroencephalogram	MAP	= Mean arterial pressure
BIS	= Bispectral index	NIRS	= Near-infrared spectroscopy
BS	= Burst suppression	PaCO_2_	= Partial pressure of carbon dioxide
cEEG	= Continuous electroencephalogram	PDA	= Patent ductus arteriosus
CLV	= Continuous low voltage	rScO_2_	= Regional cerebral oximetry
CMRO_2_	= Cerebral metabolic rate of oxygen	rSO_2_	= Regional oximetry
CNV	= Continuous normal voltage	rSsO_2_	= Regional somatic oximetry
CPB	= Cardiopulmonary bypass	SaO_2_	= Arterial oxygen saturation
EEG	= Electroencephalogram	SEF	= Spectral edge frequency
FT	= Flat trace	SjO_2_	= Jugular venous oxygen saturation
Hb	= Hemoglobin	SvO_2_	= Central venous oxygen saturation
ICP	= Intracranial pressure	SpO_2_	= Pulse oximetry
MAC	= Minimum alveolar concentration		

## INTRODUCTION

Real-time neurologic and cardiorespiratory function, central temperature, inhaled anesthetic concentrations, and renal function monitoring should be an integral part of depth anesthesia monitoring and neuroprotective strategies for pediatric patients requiring major surgery, such as cardiac and major abdominal surgeries, particularly neonates. These allow swift and appropriate management to provide appropriate anesthesia level and drug combinations and detect and counteract cerebral oxygenation decreasing. Ideally, real-time monitoring should allow reliable, reproducible, and easy tasks for detecting adverse outcome events and their causes and adequate anesthesia level.

**Fig. 1:**
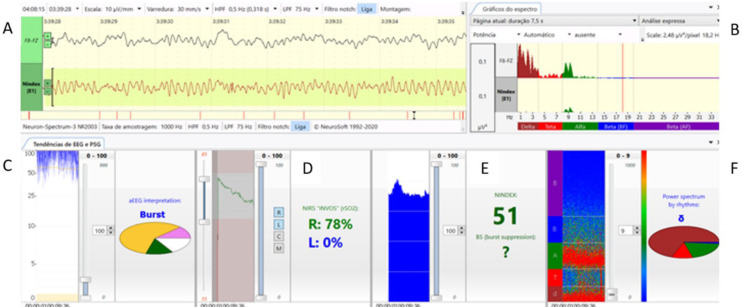
Parameters exhibited vertically in a 10-minute trend window: A) continuous electroencephalogram; B) spectral graphic (spectrum); C) amplitude-integrated electroencephalogram (aEEG); D) rScO2; E) NINDEX and F)-spectrogram, recorded at Fz-F8 channel from a two-years old patient undergoing ventricular septal defect repair.

In this article, we describe the multimodal and multiparametric Neuron-Spectrum software system (Kandel®) composed of continuous electroencephalogram (cEEG), spectral graphic (spectrum), spectrogram, amplitude-integrated electroencephalogram (aEEG), NINDEX (anesthesia depth monitor), and regional oximetry provided by near-infrared spectroscopy (NIRS) method (INVOS™), that we recently have established as a standard of care for pediatric patients undergoing cardiac and major non-cardiac surgeries to monitor anesthetic effects, cerebral oxygen supply, and cortical functional integrity (Figure 1).

## MEASUREMENT OF CEREBRAL OXYGENATION/REGIONAL OXIMETRY (NIRS)

The most frequently employed technique is the determination of regional oxygen saturation (rSO_2_) by NIRS and jugular venous oxygen saturation (or SjO_2_), representing regional and global (hemispheric) oxygenation. These two variables are highly correlated^[1,2]^, describing the relation between cerebral metabolic rate of oxygen (CMRO_2_) and cerebral oxygen supply. NIRS has the advantage of being non-invasive and a dynamic marker in real-time, without requiring pulsatile signals, like pulse oximetry. This is especially useful during cardiopulmonary bypass (CPB) with hypothermia and during resuscitation after a cardiac arrest. The cerebral NIRS provides a reliable measure of venous saturation below the sensors, especially in small skulls such as those from neonates^[3]^.

**Fig. 2:**
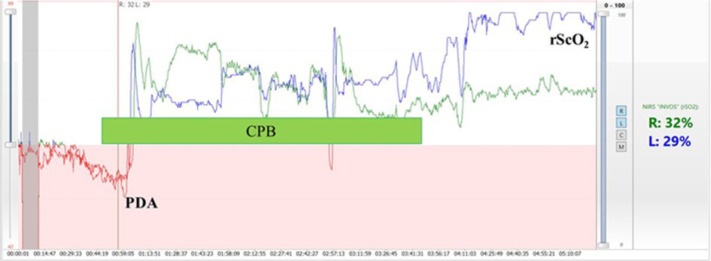
Neonate cerebral/somatic regional oximetry (rScO2/rSsO2). Recovery of the desaturation after interruption of the pulmonary runoff by patent ductus arteriosus (PDA) closure during a Jatene surgery. The rScO2 behaviors at the end of the surgery might indicate an increase in the cerebral blood flow due to cerebrovascular autoregulation disruption and increase in blood pressure. CPB=cardiopulmonary bypass; rScO2=regional cerebral oximetry

The rewarming period during CPB and persistent hypotension during anesthesia carry the greatest risk for cerebral hypoxia regardless of appropriate arterial oxygen saturation (SaO_2_)^[4]^. As a single factor, regional cerebral oxygen saturation (rScO_2_) has the highest likelihood of detecting conditions associated with cerebral hypoxia. Cerebral desaturation may occur requiring aggressive management, but neither peripheral arterial oxygen saturation (SpO_2_), mean arterial pressure (MAP), or central venous oxygen saturation (SvO_2_) can predict it. In order to keep normal rScO_2_ (> 60%), a significant variation in the interactions between cerebral oxygen consumptions (central temperature, anesthesia depth), blood flow runoff (pulmonary shunt), arterial oxygen concentration (SaO_2_), arterial partial pressure of carbon dioxide (PaCO_2_), MAP and a wide variation in CPB flow, vasoactive drugs, and volume administration may be necessary. The increase of rScO_2_ associated with arterial hypertension may indicate an alteration in cerebrovascular autoregulation induced by hypothermia during CPB^[2,5]^ (Figure 2).

## FUNCTIONAL MEASUREMENT OF BRAIN ACTIVITY AND INTEGRITY

### Electroencephalogram (EEG) and Spectrum (Spectral Graphic)

During the perioperative period of pediatric surgery, EEG monitoring permits the detection of neocortical synaptic activities. In addition to cerebral perfusion, the EEG pattern is influenced by cerebral temperature, alert state, anesthetic drugs, and age. EEG signals may suffer electric interferences and its interpretation is operator-dependent. However, some algorithms can be applied to reduce their complexity and the interpretation task. Visual analysis of EEG trace (voltage oscillations, µV), used by neurologists to access epilepsy or sleep disturbs, requires attention, experience, training, and is time-consuming, usually not suitable during anesthesia for cardiac surgery. Automated EEG analysis in time domain measures the amplitude (µV), frequency (Hz), and burst suppression (BS) rate. In frequency domain, spectral analysis process of fast Fourier transform decomposes the EEG oscillations (signals) to specifics physiologic amplitude and frequency bands: gamma (γ) consists of very rapid oscillations (> 30 Hz), associated consciousness, and perception; beta (β) (13-30 Hz) presents in patients awake and alert; alpha (α) (8-13 Hz) is observed in subjects relaxed with eyes closed; theta (θ) (4-8 Hz) is seen in light sleep; delta (δ) (1-4 Hz) is predominant during deep sleep, deep anesthesia, and coma; and slow (< 1 Hz). Fourier analysis of the EEG (spectral analysis) generates a series of numerical descriptors or parameters, including total spectral power (µV^2^) (overall signal variability), bands frequency relative power (µV^2^), spectral edge frequency (SEF) (90 %- the frequency under which occurs 90 % of the total power), median frequency (F50) (equal to SEF 50%), and percent of contribution for each band to total power, almost in real-time^[6,7]^.

### Spectrogram (Anesthetic EEG Signatures)

**Fig. 3:**
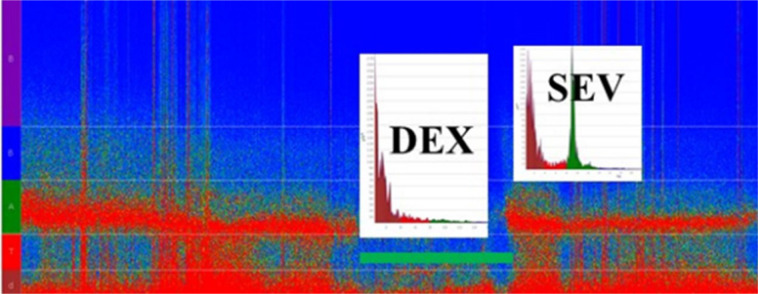
Spectrogram of a frontal channels (Fz-F8) recording during anesthesia for atrial septal defect correction of a seven-year-old patient maintained with remifentanil (0.1-0.3 µg/kg/min), dexmedetomidine (DEX) (1 µg/kg/h), and sevoflurane (SEV) (expiratory concentrations: 0.5-2.2 %). DEX corresponded to a period when no sevoflurane was administered, and a typical DEX-electroencephalogram (EEG) signature (delta frequency band dominance) could be seen. SEV (2.2 %) was given after weaning off the cardiopulmonary bypass (green line), producing the sevoflurane EEG signature (delta and alpha frequency bands dominance).

Since the spectrum or spectral graphic represents a single segment of EEG data and it is not stationary (it changes constantly), it can be computed in a successive spectrum of 3-second and 0.5-second overlap windows displayed as a spectrogram, which is an EEG activity tendency overtime displayed in the two-dimensional plot (frequency × time and power coded by color); it is practical and informative to readily observe a change of the EEG in time to an specific anesthetic dosing, called drug-specific signature, and balance of analgesia and arousal-provoking stimuli. For example, the two GABA-A agonists sevoflurane (at 1 minimum alveolar concentration [MAC]) and propofol (at hypnotic plasma concentrations) have the EEG similar signatures of slow-delta and alpha oscillations. However, sevoflurane > 1 MAC also produces theta oscillations, indicating a more profound state of unconsciousness, while dexmedetomidine EEG signature shows slow-delta and spindles that resembles stage II of non-rapid eye movement sleep^[6,8]^ (Figure 3). Today, most brain monitors of anesthesia depth available in Brazil (bispectral index [BIS], NINDEX, and SedLine®), besides their specific indices, display the continuous EEG and the spectrogram (specific anesthetic EEG signature) that allows tracking the changes in anesthesia states and management.

### Depth Anesthesia Monitor (BIS, NINDEX)

BIS quantitate the cortical activity by spatial non-coherence presents a probabilistic relation with the depth of anesthesia and sedation, however, it is influenced by various drugs, temperature, and brain supply of oxygen and glucose. During cardiac surgery, besides general anesthetics routinely used in high doses (opioids and midazolam), profound hypothermia (< 28º C) may depress the EEG to an isoelectric point indistinguishable from that produced by profound ischemia. Other pathological factors, such as hypoglycemia, hypocapnia, hypocalcemia, and hyponatremia can profoundly affect EEG. The EEG can detect cerebral hypoxia but is significantly less sensitive than NIRS in detecting more subtle brain oxygenation^[9,10]^.

On CPB, processed EEG technology monitoring, such as BIS or NINDEX, is advantageous for anesthetic depth determination, since usual clinical signs of anesthesia are not available. BIS monitoring computes a number that ranges from mean awake values of 90-100 to zero (isoelectric) in adults, children, and infants. During hypothermia, BIS values are reported to decrease, reducing the anesthetic dose required to achieve BIS values of 40-60 (deep hypnosis levels). BIS monitoring is reported to help the detection of central nervous system hypoperfusion and cerebral embolism. BIS value is reported to increase during rewarming phase, perhaps reflecting higher conscious levels. Combined with NIRS, BIS helps to detect cerebral ischemia during cardiac surgery in children^[9,11]^, and in adult cardiac surgery, anesthesia controlled by BIS monitoring may facilitate anesthetics agents titration, decreasing hemodynamics disturbances^[11]^.

EEG changes with brain maturation, it is quite fast in the first year of life and becomes like an adult after one or two years of age. Some specific features of pediatric EEG include slow and delta oscillations and spindles and discontinuity or trace alternant predominance in the neonate awake state. The dominant frequency and beta ratio increase with age, at 3-5 months of age it is 4-6 Hz and reaches 8-9 Hz at three years of age. The total EEG power reaches a peak at eight years of age. These EEG differences throughout infancy imply in developing age-dependent strategies to monitor children’s brain state properly, particularly neonates receiving general anesthesia. So that, BIS has low performance in predicting arousal during propofol, and remifentanil and inhaled anesthesia^[12-14]^ have a poor correlation with sevoflurane concentrations in infants (< 1 year of age)^[14]^. Therefore, in our practice, we record the anesthesia depth index with the NINDEX in children older than one year. NINDEX is an anesthesia depth monitor software incorporated in the Neuron-Spectrum system. Like BIS, it has a proprietary algorithm that generates non-dimensional values 0-100, and the simultaneous reading of anesthesia depth runs in parallel with BIS, but with higher values than BIS during maintenance of anesthesia and some differences during induction and emergence of anesthesia (own observations). Perhaps, in infants, lower frequency components (low, delta, and theta bands in the spectrogram) and F50 can be useful to discriminate unconsciousness/awake states and predict arousal during general anesthesia^[12]^.

### aEEG (Amplitude-integrated EEG)

An innovative combination of NIRS and aEEG provides measures of the cerebral oxygenation, hemodynamic, and cerebral electrical function in response to cardiorespiratory intervention and anesthetic drugs^[15]^, particularly in infants in whom BIS is not a reliable monitor of the depth of anesthesia.

The aEEG is a simplified method for brain function monitoring that displays trended brain activity. It is widely used as a monitoring tool for cerebral activity in the neonate in intensive care unit. It offers continuous long-term monitoring of electrical brain activity, which access the background activity and detect the sleep-awake cycling and seizures. The raw cEEG is reported to be useful for depth anesthesia and brain functional integrity monitoring and can allow early prediction of neurological outcome, particularly in neonates with hypoxic-ischemic encephalopathy. The continuous display of the spectral graphic and anesthesia depth index (BIS or NINDEX) and SEF (95 or 90%) or median frequency (F50) can also provide indications of anesthesia depth and adequacy and cerebral functional integrity. It may also detect epileptic discharge and signs of hypoxia-ischemia^[16,17]^. However, special training is required for interpreting EEG findings. The aEEG is a quantitative processing derived from single or dual-channel cEEG recordings. aEEG findings can be easily interpreted by non-neurologists^[16,17]^. cEEG and aEEG are the best methods for detecting seizure activity which may be subclinical in manifestation or obscured by neuromuscular blockade^[18]^.

**Fig. 4:**
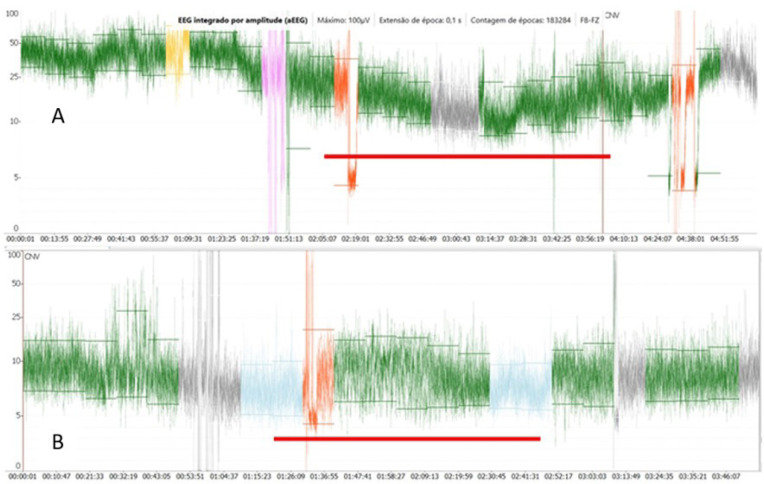
Two frontal channels aEEG complete recordings from two patients with general anesthesia maintained with sevoflurane, remifentanil, and dexmedetomidine: A) Five-months and 20-days of age patient undergoing atrioventricular canal defect repair; B) one-month and 25-days of age patient undergoing Tetralogy of Fallot repair. The amplitudes (maximum, minimum, and average) and the background patterns trends are appropriate for their ages. However, the upper trace have higher amplitudes and less variations that may reflect the cerebral maturation during the first year of life.

In our practice, EEG signals are recorded from one or two frontally placed electrodes corresponding to Fz (reference electrode), F8, or F7 (active electrodes), according to the international EEG 10-20 classification. The montage consisted of two channels: Fz-F8 and Fz-F7. The NINDEX montage consisted of EP1 (reference) and A1 (preauricular active electrode). The signal is amplified and filtered to activity < 2 Hz and > 15 Hz to minimize artifacts from sweating, movement, muscle activity, and electrical interference. The processed signals are presented on a semilogarithmic scale, rectified, smoothed, and time-compressed (6 cm/h). The bandwidth reflects the variation in maximum and minimum EEG amplitude. So, aEEG represents graphically the variation tendency, peak-to-peak, of EEG’s amplitude in a long period. The voltage activity is classified by voltage criteria (minimum, maximum, and average) (µV) and by background pattern recognition and classified in the following categories: continuous normal voltage (DNV), upper margin of ≥ 10-25 µV in term infants and lower margin of 5-10 µV; discontinuous normal voltage, lower margin < 5 µV and upper margin > 10 µV; BS, discontinuous trace with a lower margin between 0-2 µV punctuated by burst activity with amplitude > 25 µV; continuous low voltage (DLV), low amplitude trace with upper margin ≤ 5 µV and burst < 25 µV; and flat trace (isoelectric) (FT), traces without burst with amplitude < 5 µV^[17,18]^ (Figure 4).

**Fig. 5:**
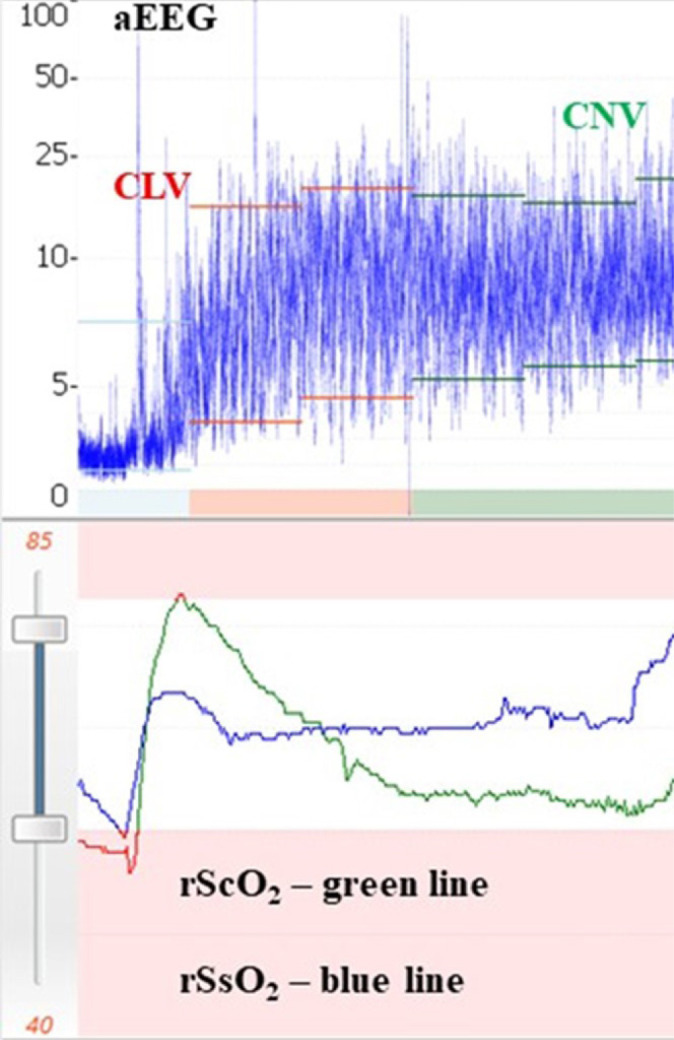
Recovery of cerebral (rScO2) and somatic (rSsO2) regional oximetry in a neonate after cardiovascular arrest during Transposition of Great Arteries repair with simultaneous electrophysiologic recovery accessed by amplitude-integrated electroencephalogram (aEEG). CLV=continuous low voltage; CNV=continuous normal voltage.

Some clinical applications include determination of brain maturation, background patterns, and sleep-awake cycles, examining the effects or impacts of sedation and anesthesia on the pre-term infants, seizure detection, and therapeutic monitoring^[19,20]^ to determine the eligibility for therapeutic hypothermia in a neonate with hypoxic-ischemic encephalopathy^[20]^. The pediatric values of aEEG (background patterns) have been used for prediction of neurological outcome in neonates diagnosed with hypoxic-ischemic encephalopathy and pre-term infants with intraventricular hemorrhage, after major cardiac and non-cardiac surgery in the neonatal period^[18,21]^. In the neonate, the cerebral oxygen supply recovery measure by regional oximetry (rScO_2_) after a cardiovascular arrest, with simultaneous recovery of functional activity as measured by aEEG, can be a reliable indication of adequate neuroprotection (Figure 5).

EEG-derived monitors, or processed cEEG, are not frequently employed to investigate the depth of anesthesia in pediatric patients, particularly neonates^[14]^. Many anesthesia depth monitors interpret EEG frequency and time domains^[22]^, whilst others evaluate EEG’s entropy^[23]^. The aEEG is obtained from cEEG, reflects peak-to-peak EEG fluctuations, and is obtained using an enveloped detection algorithm^[24]^. EEG characteristics of infants are quite different from older children and may explain why BIS monitoring tool to guide anesthesia depth in neonates is not appropriate^[24]^. General anesthesia in neonate produced variable and transient reduction on brain activity accessed by aEEG. After cessation of anesthesia, 78% recover the pre-anesthesia background pattern within one hour^[24]^. The dose or concentration of sevoflurane is associated with the level of reduction in brain activity. However, in children younger than two years of age, aEEG mean amplitude was not correlated to multiple sevoflurane MAC. On the other hand, BIS has some correlation in children older than one year of age. Nevertheless, the background patterns can be a useful indicator of depth anesthesia in infants rather than amplitude alone. During anesthesia in term neonates, the background activity may be reduced by one or two levels or remain constant (CNV) without burst activity^[14]^.

Early brain activity is essential for neuronal development^[25]^. Anesthesia causes relatively short depression of brain activity. One of the first cohorts calls attention to decreased language, comprehension and performance in children exposed to anesthetics before four years old^[26]^. Worldwide concerns have been raised on the potentially harmful effects of general anesthesia on a young infant’s brain. Food and Drugs Foundation and American Academy of Pediatrics have recommended reducing young children’s overall drug dosage. Probably, monitoring the brain activity by a processed EEG during anesthesia could ensure adequate dosing, reduce the risk of awareness, and shorten recovery^[27]^.

## CONCLUSION

**Fig. 6:**
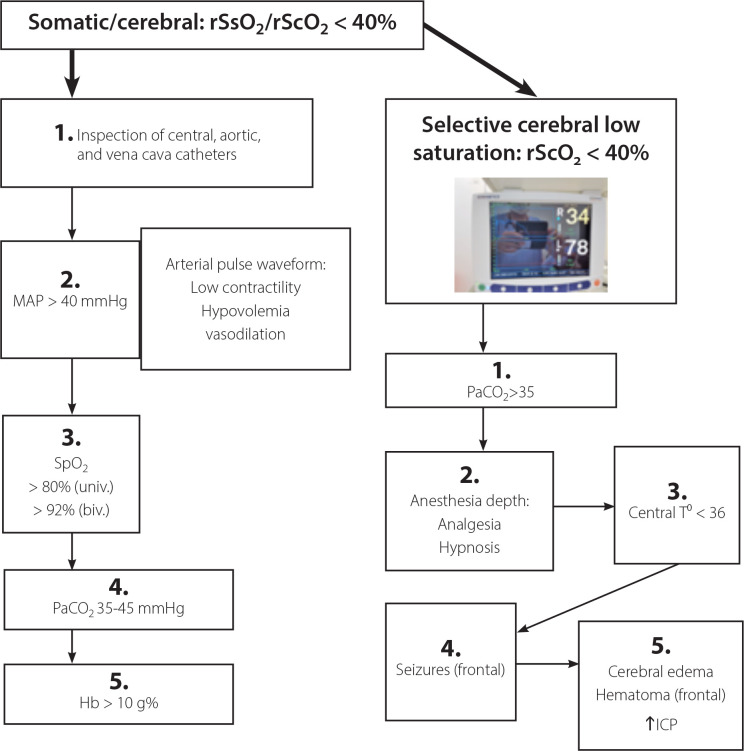
Physiologic algorithm approach to reversal cerebral oxygen desaturation during pediatric cardiac surgery. A) Cerebral/somatic desaturation (rScO2/rSsO2< 40%). Step 1 - verify the positions of central, arterial, and vena cava catheters; Step 2 - adjust mean arterial blood pressure within 20% of the pre-induction levels or > 40 mmHg for neonates and > 50 mmHg for infants. Diastolic and systolic arterial pressures and arterial pulse waveform may guide volume, inotropic, and vasopressor drugs administration; Step 3 - adjust the ventilation parameters, increase the fraction of inspired oxygen, and reduce the pulmonary vascular resistance; Step 4 - correct hypocapnia (PaCO2< 30 mmHg) to obtain a PaCO2 of 35-45 mmHg; and Step 5 - consider giving blood transfusions if hemoglobin (Hb) < 10 g% or hematocrit < 30%. B) If selective cerebral hypoxia ensues after correction the systemic hypoperfusion and oxygenation. Step 1 - correct hypocapnia; Step 2 - increase the anesthesia depth (hypnotics) and analgesia (opioids). Anesthesia electroencephalogram (EEG)-based monitor such as BIS (40-60) or NINDEX (values between 50-70) can be useful in children > 1 year of age, but in infants, predominance of slow-delta power observed in the spectrum and spectrogram (EEG anesthetics signature) and lower background of amplitude-integrated EEG are more reliable. Step 3 - avoid hyperthermia (central temperature > 37 C⁰ and decrease to less than 35⁰ C to reduce cerebral oxygen consumption and perform neuroprotection; Step 4 - if a seizure is detected, it will be treated with midazolam, which is normally used during cardiac surgery; Step 5 - consider neurologic assessment and magnetic resonance imaging or computed tomography scan to access cerebral edema or hematoma and intracranial hypertension. Hypoglycemia, hypocalcemia, and hyponatremia should be monitored since they may cause significant EEG depression. ICP=intracranial pressure; MAP=mean arterial pressure; SpO2=pulse oximetry; univ (univentricular); biv (biventricular)

wwNowadays, due to practical reasons, NIRS is the most frequently brain monitor used and it has been adopted as the endpoint in many algorithms^[28]^ for hemodynamic and respiratory management during cardiac surgery to improve patient outcomes. Figure 6 shows an algorithm based on cerebral and somatic regional oximetry currently used in our routine. Various continuous monitoring modalities, such as regional oximetry (NIRS) and processed EEG (BIS or NINDEX), and physiological monitoring are complementary rather than exclusive. In our neuromonitoring system, the cEEG, spectral graphic (spectrum), aEEG, NINDEX (anesthesia depth index), rScO_2_/rSsO_2_, spectrogram (a tendency of spectral frequencies - Neuron-Spectrum, Kendal), and SEF (50%) are displayed and recorded in the same time frame, so the immediate (on real-time) synchronized trend curves can provide more useful clinical information. The placement of EEG electrodes and INVOS™ sensors is time-consuming, so a sensor with the electrodes built-in would reduce time and increase the quality of the monitoring. However, employing the monitoring pattern here described one should detect cerebral hypoxia-ischemia and loss and recovery of consciousness, establish the adequacy of surgical anesthesia at various ages range (neonates, infants, and children) and allows identification of cerebral drugs effects.

**Table:** 

Authors' roles & responsibilities	
JGK	Substantial contributions to the conception of the work; and acquisition, analysis, and interpretation of data for the work; drafting the work; final approval of the version to be published
WNPG	Substantial contributions to the conception of the work; and acquisition, analysis, and interpretation of data for the work; drafting the work; final approval of the version to be published
MC	Substantial contributions to the acquisition of data for the work; final approval of the version to be published
LVG	Substantial contributions to the acquisition of data for the work; final approval of the version to be published
ACM	Substantial contributions to the acquisition of data for the work; final approval of the version to be published
